# Proposal of a Rapid Detection System Using Image Analysis for ELISA with an Autonomous Centrifugal Microfluidic System

**DOI:** 10.3390/mi15111387

**Published:** 2024-11-16

**Authors:** Shunya Okamoto, Yuto Mori, Shota Nakamura, Yusuke Kanai, Yoshiaki Ukita, Moeto Nagai, Takayuki Shibata

**Affiliations:** 1 Department of Mechanical Engineering, Toyohashi University of Technology, Toyohashi 441-8122, Japan; 2 Graduate Faculty of Interdisciplinary Research, University of Yamanashi, Kofu 400-0016, Japan; 3 Institute for Research on Next-Generation Semiconductor and Sensing Science (IRES2), Toyohashi University of Technology, Toyohashi 441-8122, Japan

**Keywords:** centrifugal microfluidics, POCT, ELISA

## Abstract

In this study, with the aim of adapting an enzyme-linked immunosorbent assay (ELISA) system for point-of-care testing (POCT), we propose an image analysis method for ELISAs using a centrifugal microfluidic device that automatically executes the assay. The developed image analysis method can be used to quantify the color development reaction on a TMB (3,3′,5,5′-tetramethylbenzidine) substrate. In a conventional ELISA, reaction stopping reagents are required at the end of the TMB reaction. In contrast, the developed image analysis method can analyze color in the color-developing reaction without a reaction stopping reagent. This contributes to a reduction in total assay time. The microfluidic devices used in this study could execute reagent control for ELISAs by steady rotation. In the demonstration of the assay and image analysis, a calibration curve for mouse IgG detection was successfully prepared, and it was confirmed that the image analysis method had the same performance as the conventional analysis method. Moreover, the changes in the amount of color over time confirmed that a calibration curve equal to the endpoint analysis was obtained within 2 min from the start of the TMB reaction. As the assay time before the TMB reaction was approximately 7.5 min, the developed ELISA system could detect TMB in just 10 min. In conventional methods using a plate reader, the assay required a time of 90 min for manual handling using microwell plates, and in the case of using automatic microfluidic devices, 30 min were required. The time of 10 min realized by this proposed method is equal to the time required for detection in an immunochromatographic assay with a lateral flow assay; therefore, it is expected that ELISAs can be performed sufficiently to adapt to POCT.

## 1. Introduction

Microfluidic devices, such as lab on a chip, are promising technologies for the accurate handling of minute amounts of reagents [1,2,3]. They can undergo small numbers of chemical reactions in small quantities [4,5,6,7,8,9].

In recent years, point-of-care testing (POCT) has attracted considerable attention and has been widely studied [10,11,12,13,14]. POCT is a minimally invasive, easy-to-use, and rapid technique. Therefore, POCT can contribute to reducing the burden on patients [15]. Moreover, POCT also allows patients to perform the test themselves [16]. This is expected to reduce the risk of spreading infectious diseases, such as COVID-19, by eliminating the need to go to the hospital during an outbreak [17].

The adaptation of a chemical analysis for POCT can contribute to improving people’s quality of life for the reasons mentioned above, and many studies have been conducted on analytical methods in the world to genetic analysis, immunoassays, etc., using microfluidic chips [18,19].

An enzyme-linked immunosorbent assay (ELISA) is a method of chemical analysis using an antigen–antibody reaction [20,21]. It has been used for the detection of biomarkers in blood. In a sandwich ELISA, the most common type of ELISA, antigens are captured to the immobilized antibody (capture antibody) on the microwells by an antigen-antibody reaction; then, the enzyme-labeled antibody (secondary antibody) is allowed to react with the antigen, and antigens are then detected and quantified by reacting the substrate with the enzyme [22]. Therefore, the dynamic range is generally adjusted according to the concentration of the enzyme-labeled antibody and the reaction time of the substrate. The substrate is neutralized by adding a reaction stop solution (for example, sulfuric or phosphoric acids) after a suitable time to stop the enzymatic reaction, and the optical density (OD) is measured to quantify the amount of substrate color development (signal).

If the ELISA is adopted for POCT, it is desirable to be able to analyze it in as short a time as possible [23]. In addition, considering the disposal issues, it is desirable to reduce the amount and type of reagents required. In this paper, we propose an image analysis method for the substrate color-developing reaction that does not require a reaction stopping solution and enables rapid analysis by measuring the amount of the substrate color-developing reaction immediately after the start of the reaction.

The reaction time for this substrate is usually adjusted in advance according to the range to be measured. For example, long reaction times are required if a low concentration is measured. However, this increases the labor involved. In addition, depending on the environment, such as the temperature at the site, the same reaction time may not yield the desired amount of color development and may fall outside the measurement range. Because the proposed method continuously quantifies the development of color, this prior adjustment is unnecessary, and the reaction time can be adjusted immediately according to environmental conditions. Therefore, the proposed method can contribute to reducing the risk of an inability to measure and increasing the burden on patients.

We developed a low-cost, simple device to perform fluid control for ELISAs [24,25] and confirmed that a simple rotation control system consisting of a DC motor, an Arduino (open-source electronic platform allowing users to create interactive electronic objects), and an injection-molded chip can perform the fluid control necessary for ELISAs [26]. The device was confirmed to be capable of performing fluid control required for ELISAs using a simple rotation control measure. Thus, this device executes the most complex flow control and consists of simple components.

In previous research on rapid analysis systems, there are some reports about image analysis methods using a camera [27,28], but there are few reports on the methods for the color-developing reaction of TMB (3,3′,5,5′-tetramethylbenzidine) substrates using ELISAs. For example, Seddaoui and Amine reported a method for analyzing the yellow color of a product that was reacted with TMB and stopping reagents using a smartphone [29]. In contrast, in paper-based microfluidics, some examples [30,31] of image analysis methods focus on the blue color of the TMB reaction without using a stopping reagent. In this paper [32], a method using a centrifugal microfluidic device was reported, but there were methods for endpoint analysis that could not be applied to large chips, such as the whole surface of the CD (compact disk), due to the effect of the light source.

In this paper, we report on an image analysis method and its integration with an ELISA system for rapid detection and its application to kinetically analyze the color of the TMB substrate in a color-developing reaction. The advantages of the proposed method were evaluated by comparing the detection reactions of conventional and our proposed methods.

## 2. Materials and Methods

### 2.1. Design and Fabrication of the Centrifugal Microfluidic Chip and Observation Systems

**Figure 1:**
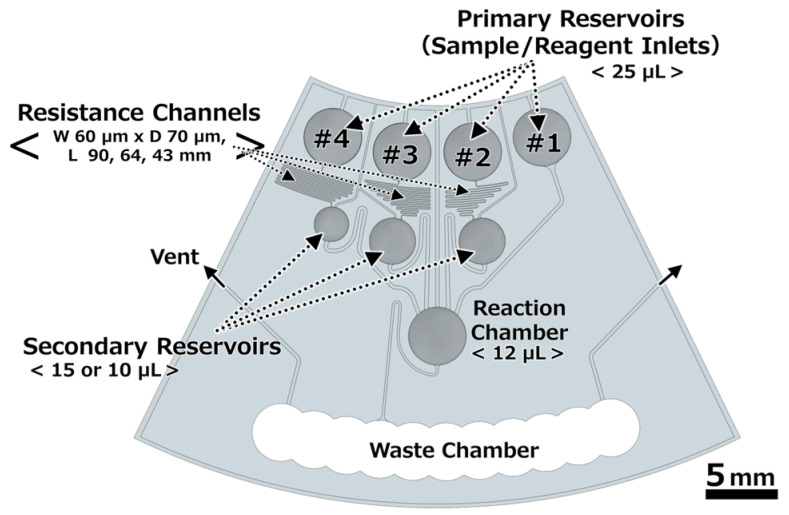
Automatic ELISA device based on CLOCK theory.

**Figure 2:**
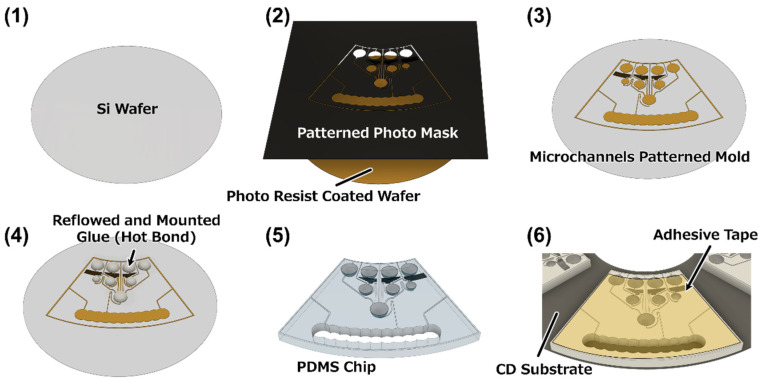
Chip fabrication process. (1) The photoresist was then spin coated onto a silicon wafer. (2) The photoresist was exposed to UV light using a micro-structured patterned photomask. (3) Development of a micropattern. (4) Reservoir shapes were created by a reflow process using glue on the mold. (5) PDMS chips were fabricated using soft lithography, and the waste chamber was punched out. (6) The microchips were mounted onto a CD substrate, and the surfaces were covered with adhesive tape without inlets of a micropipette.

Automatic ELISA devices were designed, as shown in Figure 1. These devices executed an antigen–antibody reaction, a washing process replicated twice, and an enzyme reaction by rotating steadily based on the CLOCK [25] fluidic circuit. The microchips were made of polydimethylpolysiloxane (PDMS) using a general photolithography process, a soft lithography process [33], and a reflow process [34] (Figure 2). First, a photoresist (SU-8 3050, KAYAKU Advanced Materials, Inc., Tokyo, Japan) was spin coated onto a Si wafer, followed by being covered with a photomask to pattern the channels, chambers, and reservoirs and subsequent exposure to UV light. After post-exposure baking, microchips were developed and rinsed with isopropanol. Next, the glue sticks were melted to form reservoirs. The glue sticks were adjusted to any desired volume, and each part was melted on the patterned photoresist by heating to form inlet reservoirs, secondary reservoirs, and a reaction chamber. Thus, solid hemispherical shapes were formed upon cooling to room temperature. This mold was used to form the PDMS chips. The PDMS monomer and curing reagent (SILPOT 184; Dow Corning Toray Co., Ltd., Tokyo, Japan) were mixed in a ratio of 10:1 and then applied to the mold. The PDMS chips were cured by heating at 75 °C for 90 min, and after peeling them off from the molds, the cured PDMS chips were additionally heated at 200 °C for 90 min to increase their hardness. The waste chamber was formed by punching. The PDMS chips were mounted on a CD substrate via self-adsorption. Note that the backside of the CD substrate was pasted with white tape for to visualize the liquid and color-developing reaction. Clear adhesive tape was pasted onto the chip surface to shut the reservoirs. The portions closing the ports for loading the reagents were cut using a knife.

**Figure 3:**
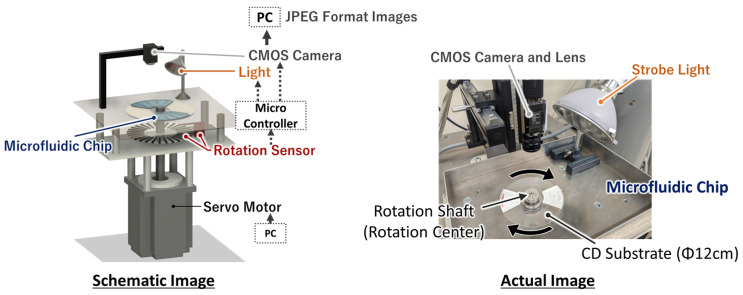
Centrifuge and observation system.

The equipment used for centrifugation and observation is shown in Figure 3. The devices were rotated by a servomotor, and images were captured by a CMOS camera (STC-SPC510PCL; Omron Sentech Co., Ltd., Ebina, Japan) using a strobe system [35] synchronized with the rotation by reading the slit of the rotating shaft. The image was sent to a PC through a camera link, and images were saved in JPEG format.

### 2.2. Preparation of Reagents for the ELISA

In the dispenser test experiment, a 0.1% (*w*/*v*) safranin (196-00032; Wako Pure Chemical Industries, Ltd., Osaka, Japan) aqueous solution was used as the working fluid. Dulbecco’s phosphate-buffered saline (DPBS) was prepared as per general composition. Bovine serum albumin (BSA; A7030-50G, Sigma-Aldrich Japan Co., LLC, Tokyo, Japan) was dissolved in DPBS at a concentration of 1% (*w*/*w*) and used as a blocking buffer, sample/standard diluent, and conjugate diluent. Tween 20 (167–11515, Wako Pure Chemical Industries, Ltd., Osaka, Japan) was added to DPBS (0.05% (*v*/*v*)) and used as a washing solution. Phosphoric acid was prepared to 1 M with ultrapure water and used as a reaction stopper for TMB (3,3′,5,5′-tetramethylbenzidine) peroxidase substrate (SeraCare Life Sciences, Inc., Milford, MA, USA, 5120-0053). To demonstrate the flow control of the multiplex ELISA device, 1% (*w*/*v*) safranin (196-00032; Wako Pure Chemical Industries, Ltd., Osaka, Japan) was dissolved in the sample/standard diluent, and 1mM fluorescein ((F6377-100G, Sigma-Aldrich Japan Co. LLC, Tokyo, Japan) was added to the wash solution to visualize the liquid. TMB reacted with a small amount of HRP and became colored.

### 2.3. On-Chip ELISA Preparation

We detected mouse IgG on the device using a commercially available ELISA Quantitation Kit (Bethyl Laboratories, Inc., Montgomery, TX, USA, E80-129). To immobilize the capture antibodies on the reaction chambers, 30 μL of anti-mouse goat antibody ((#31093, Invitrogen, Waltham, MA, USA) diluted 10-fold in DPBS was applied into the chamber and incubated for one hour at room temperature. The reaction chambers were washed three times with washing solution, vacuum dried overnight, and used for the assay. A total of 20 μL of washing solution was applied into each of the two center primary reservoirs, 15 μL of TMB solution was applied into the left-end primary reservoir, and 10 μL of standards was applied into sample/standard inlet reservoirs. Standards were prepared using a mixture of pre-reacted mouse IgG (31903, Invitrogen, Waltham, MA, USA) and HRP (horseradish peroxidase)-labeled goat anti-mouse IgG (074-1806, Kirkegaard & Perry Laboratories Inc., Gaithersburg, MD, USA) for 20 min off the chip. The reagents and standard-loaded devices were rotated steadily at 1500 rpm for 25 min. The acceleration and deceleration for approaching the target rotational speed and stopping were set to 300 rpm.

## 3. Results and Discussion

### 3.1. Effects of Light Exposure and Definition of Image Analysis Methods

One of the most significant differences between a conventional method of analysis (measuring the optical density (OD)) and an image analysis method is the method used to illuminate the sample. The conventional method is lit with a coaxial and transmitted light path, whereas the proposed image analysis method uses a lateral illumination source for a lower-cost setup. Therefore, the luminance of the image was affected by the relative position between the reaction chamber and the light source (strobe). Therefore, we first considered a method for calculating the true signal that does not affect the relative position of the CD-shaped device.

**Figure 4:**
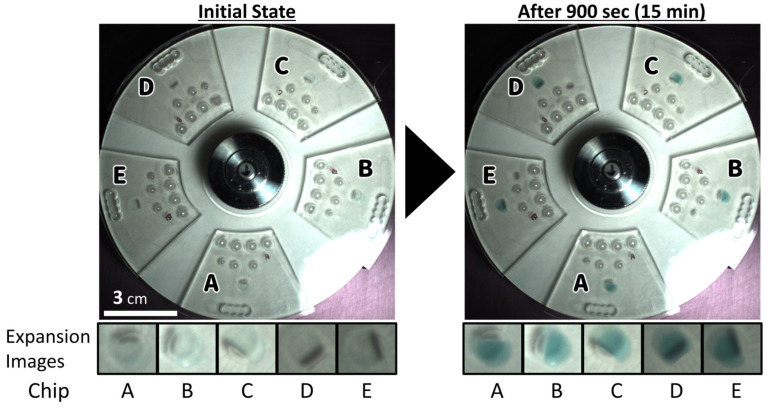
Images taken before (initial state) and after (end point) of the color-developing reaction of the TMB substrate.

In this experiment, only the enzyme reaction was carried out on five rotating chips by pre-immobilizing the HRP-labeled antibody via physical adsorption (Figure 4). The ODs at 450 nm were measured with a spectrometer after stopping the rotation and reacting with the same volume of 1M phosphoric acid; the ODs were used as the true value of the signal to evaluate image analysis.

In this study, we compared four methods for image analysis: RGB or L*a*b* color spaces and their differences. Each RGB value uses the original pixel of the JPEG-style image. The L*, a*, b*, and hue values of the L*a*b* color space [14] were converted from RGB values using Formulas (1)–(6) [36] as follows:

(1) $$\left[\begin{array}{c} X\\ Y\\ Z \end{array}\right]=\left[\begin{array}{ccc} 0.4124 & 0.3576 & 0.1805\\ 0.2126 & 0.7152 & 0.0722\\ 0.0193 & 0.1192 & 0.9505 \end{array}\right]\left[\begin{array}{c} R\\ G\\ B \end{array}\right]$$

(2) L∗=116∗fYYn−16

(3) a∗=500∗fXXn−fYYn

(4) b∗=200∗fYYn−fZZn

(5) $$h=\tan^{-1}\left(b^{*}/a^{*}\right)$$

here, note that

$$X_{n}=0.9505,\;{\;Y}_{n}=1.0,\;{\;Z}_{n}=1.089$$

(6) $$f\left(x\right)=\left\{\begin{array}{c} x^{1/3}\;x>{\left(\frac{24}{116}\right)}^{3}\\ \frac{841}{108}x+\frac{16}{116}\;x\leq {\left(\frac{24}{116}\right)}^{3} \end{array}\right.$$

**Figure 5:**
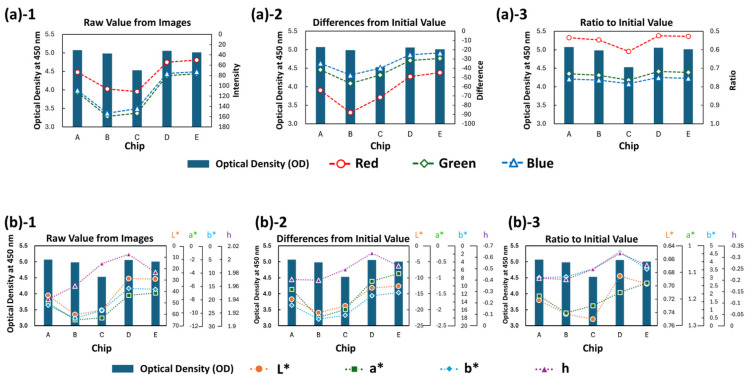
Comparison of the OD and image analysis in endpoint analysis. (**a**) RGB color space. (**b**) L*a*b* color space. (a) or (b)-1, -2 and -3 show the raw values, the differences and the ration from the initial value.

First, the measured ODs of chips A, B, C, D, and E, which were used as the true values, were 5.071, 4.984, 4.528, 5.056, and 5.011, respectively, and 4.528 for C was larger than the other values. Therefore, the analysis method satisfied the condition that the coefficient of variation (CV) of chips A, B, D, and E was less than 2%, and the same trends of larger or smaller signals were searched. Figure 5 shows the graphs of the OD (bar chart) and each image analysis value (line chart). Each row value of RGB and L*a*b*, which indicates the original value obtained from the RGB of the image without operation or the value just converted to the L*a*b* color space, did not satisfy the condition (Figure 5((a)-1,(b)-1)). It is thought that the row values affect the relative position between the strobe and the reaction chamber. However, it was confirmed that the ratio of the changed value from the initial value (when TMB was injected into the reaction chamber) satisfied the condition for each value of RGB (Figure 5((a)-3)). It is believed that the effect of light illumination is reduced by calculating the ratio at the same relative position from the light source. On the other hand, in the L*a*b* color space, lightness and darkness are a single axis (L* axis). The L* values had a correlation with the brightness of the image, and the a* and b* values also had a similar correlation (Figure 5((b)-3)). This trend was similar for RGB values as well. However, in the L*a*b* color space, the ratio of change does not correlate with OD. This suggests that the color change from transparent to blue in TMB is influenced by the lightness and darkness of the image. In other words, this color change in TMB cannot be analyzed in the L*a*b* color space using only one evaluation axis, such as L*, a*, b*, or h. In contrast, the RGB color space appears to be compatible with the color change in TMB, allowing it to be quantified using only one evaluation axis. Although it may be possible to quantify color change in the L*a*b* color space by combining multiple evaluation axes, it is thought that this approach is not desirable for low-cost and compact systems as it increases computational costs.

### 3.2. Preparation of the Calibration Curve of Mouse IgG and Rapid Detection with Image Analysis

To prepare a mouse IgG detection system using image analysis and evaluation, an ELISA was performed using the device.

**Figure 6:**
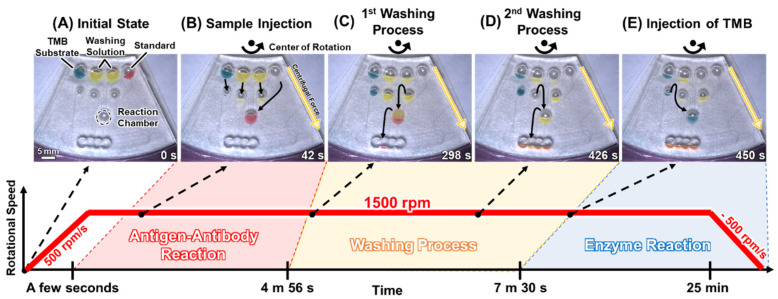
ELISA operation by the autonomously driven device.

Figure 6 shows the operation of the fabricated ELISA device using colored reagents. First, a standard solution was injected into the reaction chamber and kept in the chamber for a few seconds from the start of the rotation (Figure 6B). After approximately 5 min, the washing solution was injected into the reaction chamber and the solution and standard were drained into a waste chamber (Figure 6C). The reaction chamber was then washed again (Figure 6D) and the TMB substrate was injected into the reaction chamber (Figure 6E). Consequently, it was confirmed that the fabricated device can execute flow control for the ELISA. The flow operation movie is shown in a Supplementary Video S1.

In the assay, the concentration of the antigen (mouse IgG) was 0, 1, 10, 100, and 1000 ng/mL, and the method of image analyses used to calculate the ratio of each RGB value was evaluated by comparing the color-developed signals of TMB between the measured ODs and the results of image analysis.

**Figure 7:**
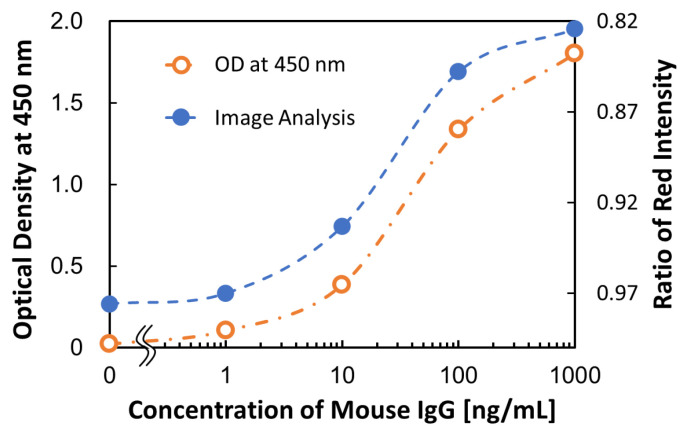
Calibration curves for mouse IgG in endpoint analysis.

Figure 7 shows the calibration curve for mouse IgG. It was confirmed that both the results of the measured ODs and the image analysis values obtained the dose–response of the concentration of mouse IgG, and these systems validated the detection system. Note that the figure shows the value of red for RGB because it had the highest coefficient of determination (R^2^ = 0.988) of the three colors. The color of the TMB substrate changed from colorless to blue upon reaction with HRP; therefore, in the RGB color space, the red value changed sensitively (decreased) and did not change the blue value. Therefore, the coefficient of determination of the red value was the highest. We developed an image analysis method for Loop-mediated Isothermal Amplification (LAMP) assays, which uses the difference in hue in a L*a*b* color space [9]. This method was also examined in the present study; however, the results were unsatisfactory. This is due to the difference in the methods used to change the color when detecting the reaction. It changes to sky blue from violet in LAMP and to blue from non-colored in the ELISA.

**Figure 8:**
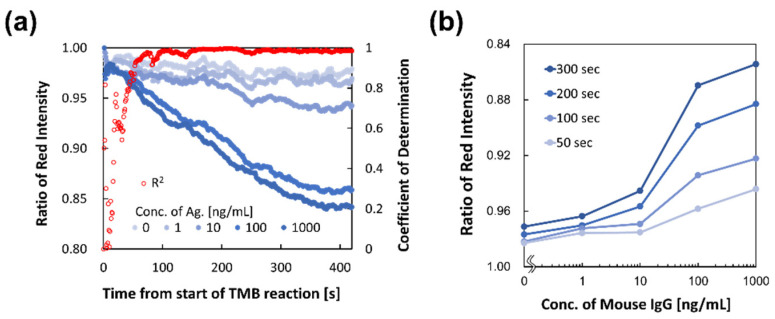
Results of kinetic analysis by the developed image analysis method. (**a**) Time-dependent change in the value of color development and coefficient determination; (**b**) calibration curves at each time interval from initiation of the TMB reaction (kinetic analysis).

We also investigated when the image analysis value was equal to the OD. Figure 8 shows a time-dependent image of the analyzed value of each antigen concentration and the coefficient of determination. The analyzed image value was affected by the flexible illumination intensity of the xenon strobe, so time-changing values were corrected by the drastically changed value (more than 5% change in a second), and the moving average was calculated for 30 s. Although the image analysis values fluctuated, the coefficient of determination exceeded 0.95 at 50 s from the start of the TMB color-developing reaction. Figure 8 shows the calibration curves for each time point from the start of the TMB reaction. The slope of the curve at 50 s was very small; therefore, it was difficult to use the curve to validate the calibration curve for this mouse IgG detection system. However, after 100 s, the slope of the calibration curve was sufficient to determine the concentration. Thus, the image analysis system can obtain a calibration curve equal to the quality of ODs in endpoint analysis within 100 s from the start of the TMB reaction. Conventional methods for measuring ODs require a reaction time of 15 min and the use of a stopping solution. Therefore, the developed image analysis method can significantly reduce the assay time. In this experiment, the time until the start of the TMB reaction was approximately 7 min and, consequently, the detection time from the start of disk rotation was approximately 8 min 40 s. This time is equal to the time required for detection in an immunochromatographic assay, and it is expected that the ELISA can be performed sufficiently quickly and accurately for POCT. It should be noted that the effect of the measurement color (wavelength) of the TMB coloring reaction between blue (OD at 620 nm) and yellow (OD at 450 nm, after reaction with stopping solution) was determined by preparing calibration curves using a microtiter plate with manual handling. T results (shown in Supplementary Information) show that the shapes of the curves were the same, but the slopes differed. This affected the sensitivity of detection, and the limit of detection (LOD) was calculated as 7.81 ng/mL with ODs of 620 nm and 0.099 ng/mL at 450 nm. Therefore, for kinetic analysis, image analysis is considered to be better than the OD measurement.

## 4. Conclusions

In this study, an image analysis method for the ELISA with a centrifugal microfluidic system was developed and validated. It was confirmed that this system was able to measure the signal of the TMB color-developing reaction by calculating the ratio of the change in the red value in the RGB color space, thereby replacing the need to measure ODs, thereby reducing assay time for detection and preparation of the calibration curve.

The fabricated devices were able to perform flow control to execute an antigen–antibody reaction and enzymic reactions for approximately 7 min. Most importantly, the time required to obtain the calibration curve was approximately 100 s. Therefore, the system could obtain a calibration curve in approximately 9 min. Conventional assays with manual handling using a microtiter plate require more than 90 min. Our new system, therefore, reduced the assay time to one-tenth that of the conventional assay.

POCT devices require minimally invasive, easy-to-use, rapid, low-cost assays. The proposed microfluidic system consists of simple chips and a centrifuge. The ELISA system based on this concept has already demonstrated that the flow operation of the ELISA can be performed with injection-molded plastic chips and a small centrifuge the size of a Bento box [21]. And then, in this paper, the analysis part was successfully developed. The camera system for image analysis can be cheap, <EUR200, because it does not require high-resolution images. Moreover, the microfluidic device can automatically operate the assay with 10 μL of sample/standard, and this volume can be collected as a blood sample from the fingertip and is, therefore, easy to use during POCT, particularly for blood analyses. On the other hand, we think that there are still some challenges to be overcome in order to realize the ELISA system applied to POCT. For example, the reported system can analyze only five samples or standards in one assay. Considering actual use, it is desirable to be able to analyze a larger number of samples so that more large-scale integration of the device is required. Moreover, the ELISA uses some wet reagents. On the other hand, most already realized POCT equipment are dry reaction systems. For this reason, the storage of reagents and the method of loading them into the chip are also issues that need to be solved. Therefore, in the future, research on solving these problems should be carried out to realize a convenient blood analysis environment.

## Data Availability

The original contributions presented in the study are included in the article, further inquiries can be directed to the corresponding author.

## Supplementary Materials

The following supporting information can be downloaded at: https://www.mdpi.com/article/10.3390/mi15111387/s1, Figure S1: Calibration curves compared by OD measurement methods; Figure S2: Photographs of device schematics and actual fabrication; Table S1: Measured Major microchannel dimensions of the fabricated device; Video S1: Flow control demonstration of the device.

## Abbreviations

ELISA	enzyme-linked immunosorbent assays
TMB	3,3′,5,5′-tetramethylbenzidine
POCT	point of care testing
OD	optical density
CD	compact disk
CLOCK	control of liquid operation on centrifugal hydrokinetics
PDMS	polydimethylpolysiloxane
CMOS	complementary metal oxide semiconductor
DPBS	Dulbecco’s phosphate-buffered saline
BSA	bovine serum albumin
HRP	horseradish peroxidase
LAMP	loop-mediated isothermal amplification
LOD	limit of detection
