# Supplementary material for: Proposal of a Rapid Detection System Using Image Analysis for ELISA with an Autonomous Centrifugal Microfluidic System
Source: Micromachines (Basel). 2024 Nov 16;15(11):1387. doi: 10.3390/mi15111387 (PMC11596746; doi:10.3390/mi15111387)
Supplement: Supplementary file 1 [file micromachines-15-01387-s001.zip › Supplementary documents.pdf]

Supplementary information

Kinetic analysis on micro titer plate assay

We carried out ELISA with manual handling using a microtiter plate, and the signal of the TMB color-developing reaction was compared between a kinetic analysis and an endpoint analysis after reaction with stopping solution using a microplate reader. The calibration curves are presented in Fig. S1. In the kinetic analysis, ODs were measured at 620 nm, and in the endpoint analysis, they were measured at 450 nm owing to the change in color upon reaction with the stop solution. The shapes of the curves were the same, but the slopes were completely different. This affected the sensitivity of detection, and the limit of detection (LOD) was calculated 7.81 ng/mL with ODs of 620 nm and 0.099 ng/mL at 450 nm. Therefore, for kinetic analysis, image analysis is considered better than OD measurement.

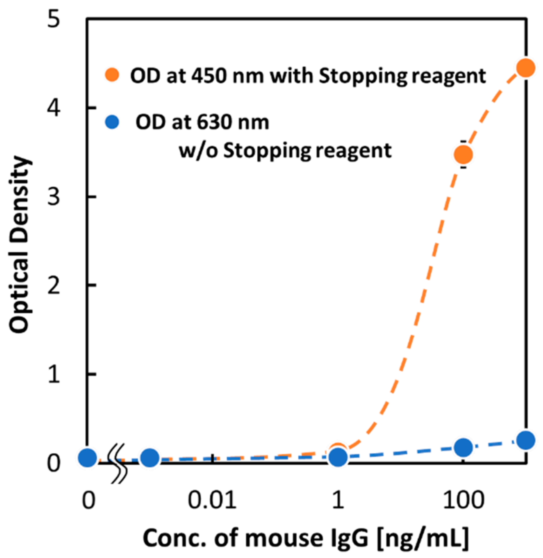

Figure S1 Calibration curves compared by OD measurement methods.

Major dimensions of the fabricated device

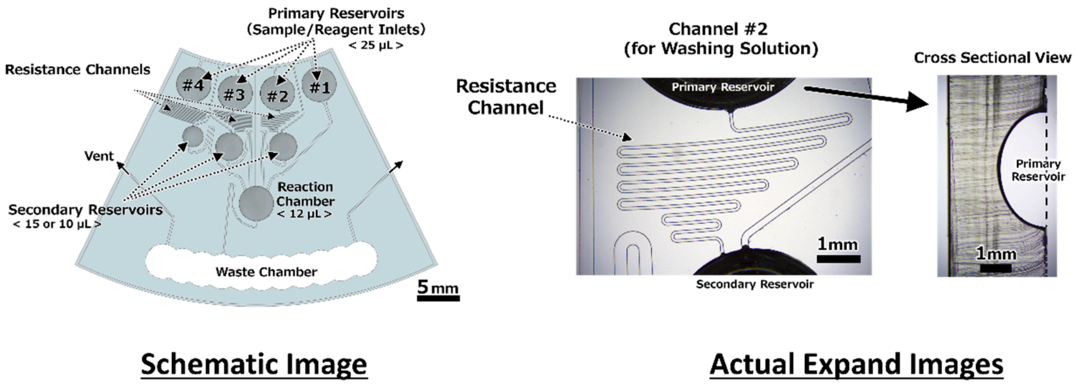

Figure S2 Photographs of device schematics and actual fabrication

Table S1 Measured Major microchannel dimensions of the fabricated device

Channel	Length [mm]	Width [µm]	Height [µm]
#1 (Sample)	17.5	296	72.1
#2 (Wash)	42.8	50.9	72.1
#3 (Wash)	64.5	52.1	75.6
#4 (TMB)	89.1	52.3	75.6
